# Bone-derived mesenchymal stem cells alleviate compression-induced apoptosis of nucleus pulposus cells by N6 methyladenosine of autophagy

**DOI:** 10.1038/s41419-020-2284-8

**Published:** 2020-02-06

**Authors:** Gaocai Li, Yu Song, Zhiwei Liao, Kun Wang, Rongjin Luo, Saideng Lu, Kangcheng Zhao, Xiaobo Feng, Hang Liang, Liang Ma, Bingjin Wang, Wencan Ke, Huipeng Yin, Shengfeng Zhan, Shuai Li, Xinghuo Wu, Yukun Zhang, Cao Yang

**Affiliations:** Department of Orthopaedics, Union Hospital, Tongji Medical College, Huazhong University of Science and Technology, Wuhan, 430022 China

**Keywords:** Macroautophagy, Apoptosis, Mechanisms of disease, Mesenchymal stem cells

## Abstract

N6 methyladenosine (m^6^A) is one of the most prevalent epitranscriptomic modifications of mRNAs, and plays a critical role in various bioprocesses. Bone-derived mesenchymal stem cells (BMSCs) can attenuate apoptosis of nucleus pulposus cells (NPCs) under compression; however, the underlying mechanisms are poorly understood. This study showed that the level of m^6^A mRNA modifications was decreased, and the autophagic flux was increased in NPCs under compression when they were cocultured with BMSCs. We report that under coculture conditions, RNA demethylase ALKBH5-mediated FIP200 mRNA demethylation enhanced autophagic flux and attenuated the apoptosis of NPCs under compression. Specific silencing of ALKBH5 results in impaired autophagic flux and a higher proportion of apoptotic NPCs under compression, even when cocultured with BMSCs. Mechanistically, we further identify that the m^6^A “reader” YTHDF2 is likely to be involved in the regulation of autophagy, and lower m^6^A levels in the coding region of FIP200 lead to a reduction in YTHDF2-mediated mRNA degradation of FIP200, a core molecular component of the ULK1 complex that participates in the initiating process of autophagy. Taken together, our study reveals the roles of ALKBH5-mediated FIP200 mRNA demethylation in enhancing autophagy and reducing apoptosis in NPCs when cocultured with BMSCs.

## Introduction

Intervertebral disc (IVD) degeneration, one of the main causes of low back pain, is becoming a great socioeconomic burden. The nucleus pulposus (NP) is a highly hydrated tissue that constitutes the inner core of the IVD. Degenerative changes in NP cells (NPCs) predominantly account for the pathogenesis of IVD degeneration. Many approaches have been applied to treat IVD degeneration, such as surgery, needle aspiration, and tissue regeneration. Many recent studies have focused on decelerating IVD degeneration by injecting or transplanting stem cells, with the aim of regenerating NP tissue with the extracellular matrix rich in collagen and proteoglycan^1,2^; the results have shown a great potential to use stem cells to reverse IVD degeneration. However, to date, the studies of the mechanisms by which stem cells aid in the restoration of the NP and disc function mostly concentrate on the differentiation of stem cells into NPCs^3,4^, but the effects of stem cell transplantation on NPCs are not fully understood.

Autophagy is a conserved cellular process to maintain homeostasis and survive under unfavorable growth conditions. Once subcellular membrane structures change, autophagosomes are formed by the encapsulation of cytoplasmic components, which then fuse with lysosomes to form autolysosomes to digest the contents. This dynamic process is called autophagic flux^5^. Autophagy has attracted increasing attention recently due to its great significance in various aspects of cell physiology, including survival during energy or nutrient limitation, and in the clearance of dysfunctional or excess organelles and proteins^6^. In addition, autophagy is associated with a range of human pathophysiological processes, including cancer^7^, cardiomyopathies^8^, and neurodegeneration^9^. Previous studies have shown that autophagy is closely involved in IVD degeneration and the degradation of the extracellular matrix in NP tissue^10,11^. Autophagy initiation starts with the activation of the ULK1 complex, which consists of ULK1 and the noncatalytic subunits FIP200 and Atg13. Several reports have demonstrated that ULK1 and FIP200 play a critical role in the induction of autophagy. Joo et al.^12^ reported that ULK1/2 conditional double KO mice, which exhibit reduced autophagic flux, showed growth retardation, premature death, and abnormal limb-clasping reflexes. Mouse embryonic fibroblasts lacking FIP200 exhibit a defect in the induction of Atg14L-Atg16-WIPI puncta upon starvation. In FIP200-deficient cells, induction of autophagy was abolished by various means, and both phosphorylation and stability of ULK1 were impaired^13,14^. Recent studies have pointed out that autophagy, an intracellular degradation process, is involved in apoptosis and could reduce apoptosis of NPCs in IVD degeneration^10,15^.

N6 methyladenosine (m^6^A) is one of the most prevalent post-transcriptional mRNA modifications in eukaryotes and plays a critical role in the regulation of the stability, translation, and translocation of a wide variety of mRNAs, and is involved in multiple cellular processes. In 2011, Jia et al.^16^ showed that fat mass and obesity-associated protein (FTO) catalyzes the process of RNA demethylation, revealing the reversibility of RNA methylation. Further studies identified a methyltransferase “writer” complex consisting of METTL3, METTL14, and WTAP that could deposit methyl residues onto the N6 atom of adenosine bases in mRNA^17,18^, and ALKBH5, an “eraser,” was found to possess RNA demethylase activity, by reversing m^6^A modifications, a function similar to that of FTO, another “eraser”^19^. Previous Me-RIP-Seq studies have shown that approximately 25% of transcripts harbor m^6^A modifications enriched around coding regions, stop codons, and untranslated regions (UTRs), and methylation at different regions may lead to different readers recognizing this process, contributing to various turnover of methylated transcripts^20,21^. After reading and binding to m^6^A-modified mRNA sites, YTHDF1 promotes translation initiation and then improves the efficiency of translation, while YTHDF2 interacts with YTHDF3 and increases the decay of modified transcripts, and YTHDC2 promotes the translation of mRNA^22–26^. A recent study has suggested that m^6^A could play a role in controlling the stability of mRNAs, as the “reader” protein YTHDF2 specifically binds to m^6^A sites and recruits the transcripts to RNA degradation complexes^22,27^. It has been shown that RNA methylation impacts several fundamental cellular processes, including the circadian clock, DNA damage repair, and meiosis^28–30^. During the processes of malignant transformation of mesenchymal stem cells and progression of breast cancer, methylation modification also has critical functions by affecting the metabolism and processing of related transcripts^31–34^. Moreover, a recent study discovered that m^6^A modification could regulate autophagy by affecting the mRNA processing of ULK1, an autophagy-related gene^35^, highlighting that this post-transcriptional modification plays a critical role in the regulation of autophagy.

In this study, we report that bone-derived mesenchymal stem cells (BMSCs) can enhance the autophagy of NPCs via m^6^A and reduce the apoptosis rate of NPCs in a coculture model. The ALKBH5-mediated demethylation of m^6^A increases the expression of FIP200, because less YTHDF2 binds to the mRNA, decelerating the degradation rate, thus promoting autophagy and inhibiting apoptosis of NPCs under compression. Our study reveals a new mechanism by which m^6^A regulates autophagy and apoptosis of NPCs, providing novel evidence for the development of BMSC injection strategies to treat IVD degeneration.

## Materials and methods

### Isolation and culture of human NPCs and BMSCs

Six human NP tissues were obtained from patients undergoing idiopathic scoliosis, which were used to isolate NP cells. Correspondingly, Pfirrmann magnetic resonance imaging-grading system was used to evaluate the level of IVD degeneration^36^. NP tissues collected from three males and three females aged 13–18 years (mean = 15.4 years) were evaluated as Grade II. The previous procedure was used to transport NP tissue, isolate, and culture NP cells^37^. FACS was used to identify NPC markers (CD24, 311117; KRT18, 628404; Biolegend, San Diego, CA, USA) with cells from the second passage. In our in vitro experiments, NPCs were exposed to an equal volume of culture medium cocultured or not with BMSCs under 1.0-MPa compression (hereafter referred to as CPR in all figures and figure legends) for 36 h. For autophagy inhibition, NPCs with or without compression were treated with medium containing Chloroquine (CQ, 100 μM, S4157, Selleck) or 3-MA (10 μM, HY-19312, MCE), and then harvested for subsequent analysis. To knock down FIP200, ALKBH5, and YTHDF2, cells were transfected for 48 h with 100 nM small-interfering RNA (siRNA) against FIP200, 200 nM siRNA against ALKBH5, and 200 nM siRNA against YTHDF2 or scrambled siRNA (GeneChem, Shanghai, China) using Lipofectamine 2000 (Invitrogen), and immediately placed under compression. In addition, the YTHDF2-FLAG plasmid was constructed and purchased from Construction Manual (GeneChem), and transfected into human NPCs following the manufacturer’s instructions. Transfection efficacies were measured by Western blotting, and the cells were further cultured for 2 days, and then passaged for the next experiment.

Human bone marrow specimens were harvested from the iliac crests of healthy volunteer donors. Isolation, expansion, identification of the surface marker, and three lineage differentiations of BMSCs were conducted as described before^37^. The cells from the second passage were cultured in Transwell inserts (Corning, 3419, New York, USA) and cocultured with NPCs (at a ratio of NPCs:BMSCs = 1:1) seeded in the lower chamber in a 100-mm dish.

All experimental protocols involving harvesting specimens and isolating cells were approved by the Ethics Committee of Tongji Medical College, Huazhong University of Science and Technology.

### Western blot analysis

Western blot procedure was performed as previously described, and GAPDH was used for normalization^37^. The following antibodies were used: anti-BCL2 (CST, #15071S, 1:1000), anti-Caspase-3 (CST, #9662, 1:1000), anti-BAX (CST, #2774, 1:100), anti-GAPDH (Affinity, AF7021, 1:2000), anti-ATG7 (Abcam, ab133528, 1/10,000), anti-Beclin1 (CST, #3738, 1:1000), anti-P62/SQSTM1 (Proteintech, 18420-1-AP, 1:1000), anti-LC3 (Abcam, ab48394, 1:2000), anti-METTL3 (Abcam, ab195352, 1:1000), anti-METTL14 (Proteintech, 26158-1-AP, 1:1000), anti-WTAP (Sigma, HPA010549, 1:1000), anti-FTO (CST, 14386S, 1:1000), anti-ALKBH5 (Abcam, ab195377, 1:1000), anti-FIP200 (CST, #12436, 1:1000), anti-YTHDF2 (Proteintech, 24744-1-AP, 1:5000), and anti-FLAG (Vigene Biosciences, FH880803, 1:1000). Horseradish peroxidase (HRP)-conjugated secondary antibodies (CST) were used, and protein bands were visualized and detected by using a ChemiDoc-It 610 Imaging System (UVP, Upland, CA, USA). The experiments were performed in triplicate.

### RT-qPCR

TRIzol reagent (Invitrogen) was used to extract total RNA from cultured cells, reverse-transcribed, and RT-qPCR was also used according to the manufacturer’s instructions. The primers used for RT-qPCR were as follows: Homo *FIP200*, forward 5′-GGAGGAGAATGCATGGCTGCAGA-3′, reverse 5′-ACTTCCTGACCAAAGATTCCACA-3′; *FIP200*-3′UTR, forward 5′-GCCCTCGGCGTTGCCTCAGAA-3′, reverse 5′-ACCATCTTCCG CCGCCGCCTA-3′; *FIP200*-CDS, forward 5′-AAGCTTTGCTCCGCCTCGTAA-3′, reverse 5′-GCTTCCTTCAACGCAAGTTCA-3′; *FIP200*-5′UTR, forward 5′-GGGCATACCTTGTGCA TTGTG-3′, reverse 5′-CCCAGATGACCAATCCACTGA-3′; Homo *METTL3*, forward 5′-CTATCCA GGCCCACAAGAAGC-3′, reverse 5′-GACACAGCATCAGTGGGCAAT-3′; Homo *METTL14*, forward 5′-CTCCTCGCGCAGCAGTTGGGA-3′, reverse 5′-TTTTGCATTTGGAGCAGAGG T-3′; Homo *WTAP*, forward 5′-AGGTTCGATTGAGTGAAACAG-3′, reverse 5′-CTTGTTCC TTGGTTGCTAGTC-3′; Homo *FTO*, forward 5′-GCTTGAAGACACTTGGCTCCC-3′, reverse 5′-GCCTTGGATCCTAACCAGGTC-3′; Homo *ALKBH5*, forward 5′-GCCGCCGAACCTTA CCCTGTG-3′, reverse 5′-CCTTCTCAGCGCGGGACACCA-3′; Homo *YTHDF2*, forward 5′-AGCCATGCCCTACTTAACTTC-3′, reverse 5′-CAGTTTAGGTTGCTGTTTTGC-3′; Homo *GAPDH*, forward 5′-TCAAGAAGGTGGTGAAGCAGG-3′, reverse 5′-TCAAAGGTGGAGGA GTGGGT-3′. GAPDH was used for normalization, and experiments were performed in triplicate.

### Immunofluorescence analysis

Immunofluorescence analysis was performed as previously described^37^. First, 4% paraformaldehyde was used to fix NPCs, and then 0.5% Triton X-100 in PBS was used to permeabilize. The slides were washed in PBS and blocked with 2% bovine serum albumin (BSA) in PBS for 2 h at 37 °C, and then incubated with anti-FIP200 (1:50) (Proteintech) for 10 h. After washing twice, the slides were then incubated with goat anti-rabbit antibody (CST) at 37 °C for 1 h. Nuclei were then co-stained with 0.1 g/ml DAPI (Beyotime, Nantong, China), and images were captured under a microscope (Olympus, BX53; Melville, NY, USA).

### TUNEL staining

For terminal deoxynucleotidyl transferase dUTP nick-end labeling (TUNEL) staining, 4% paraformaldehyde was first used to fix NPCs, and then 0.5% Triton X-100 in PBS was used to permeabilize. After washing with PBS three times, the cells were incubated with the Cell Death Detection Kit (Beyotime, Nantong, China), and nuclei were co-stained for 5 min with 0.1 g/ml DAPI (Beyotime). Then a microscope (Olympus, BX53; Melville, NY, USA) was used to capture the images.

### Transmission electron microscopy

In all, 2.5% glutaraldehyde (Sigma-Aldrich, USA) was used to fix cell samples for 1 h, and then fixed for 2 h in 2% osmium tetraoxide, washed with water, and stained with 0.5% uranyl acetate for 12 h. After dehydration and polymerization, samples were cut as ultrathin sections of 70–90 nm with an ultramicrotome, (EM UC7, Leica), and then a Tecnai G2 TWIN transmission electron microscope (FEI, USA) was used to view and capture images.

### RNA interference

Knockdown of *FIP200*, *ALKBH5*, and *YTHDF2* in NPCs was realized by transfection with siRNA. siRNA against *FIP200* (siFIP200), *ALKBH5* (siALKBH5), *YTHDF2* (siYTHDF2), and scrambled siRNA (si-NC) were synthesized by JTS Scientific (Wuhan, China) and transfected with Lipofectamine 2000 (Invitrogen) according to the standard protocol. The siRNA sequences were as follows: siFIP200, 5′-CCUAAUGAUGUGGAAUCUUTT-3′ and 5′-AAGAUUCCACAUCAUUUAGGTT-3′. siALKBH5, 5′-UCGGCUGCAAGUUCCAGUUTT-3′ and 5′-AACUGGAACUUGCAGCCGATT-3′. siYTHDF2, 5′-GCACAGAAGUUGCAAGCAAUG-3′ and 5′-UUGCUUGCAACUUCUGUGCUA-3′. si-NC, 5′-UUCUCCGAACGUCACGUTT-3′ and 5′-ACGUGACACGUUCGGAGAATT-3′. After verified high silencing efficiency, the NP cells were then used in the following treatment group.

### LC–MS/MS

The Dynabeads mRNA Purification Kit (Invitrogen) was used to purify mRNA from the total RNA. About 500 ng of purified mRNA was incubated with nuclease P1 (0.5 U, Takara) in a 25-μl reaction system containing 10 mM NH_4_OAc at 42 °C for 1 h, followed by addition of NH_4_HCO_3_ (1 M, 3 μl) and ALP (1 μl, 1 U/μl, Solarbio) and incubation at 37 °C for 2 h. After neutralization by 1 μl of HCl (3 M), samples were diluted to 50 μl and filtered with a 0.22-μm filter. All samples (10 μl for each injection) were analyzed by an Orbitrap HR-liquid chromatography–mass spectrometry (LC–MS) (Thermo Fisher). All nucleosides were quantified by use of retention time and ion mass transitions of 268.0–136.0 (A), 245.0–113.0 (U), 244.0–112.0 (C), 284.0–152.0 (G), and 282.1–150.0 (m^6^A). Ratios of m^6^A to AUCG and m^6^A to A were calculated based on calibration curves.

### Dot blot

The Dynabeads mRNA Purification Kit (Invitrogen) was used to purify mRNA from the total RNA. After 5 min of denaturation at 70 °C, equal amounts of serially diluted mRNA were added to an Amersham Hybond N+ membrane (Millipore) and cross-linked to an auto-cross-linker three times under auto-cross-linking mode. One membrane was stained with methylene blue as loading control, while the other membrane was inducted with an anti-m^6^A antibody (CST) overnight (4 °C) after blocking. The membrane was washed according to the standard protocol, and incubated with HRP-conjugated goat anti-rabbit immunoglobulin G (Proteintech, 1:1000) for 1 h. Membranes were then developed with a DAB kit (Boster, Wuhan, China) to detect the signal.

### m^6^A colorimetric assay

The Dynabeads mRNA Purification Kit (Invitrogen) was used to purify mRNA from the total RNA. EpiQuik m^6^A RNA Methylation Quantification Kit (colorimetric; Epigentek, P-9005-48) was used to measure the change of m^6^A levels in mRNA according to the manufacturer’s protocol after RNA quality analyzing by NanoDrop 2000. Poly-A-purified RNA (200 ng) was used for analysis of each sample. The m^6^A levels colorimetrically were quantified by reading the absorbance at a wavelength of 450 nm, and then relative levels were calculated based on the standard curve.

### RIP-RT-PCR

A previously described procedure was used for RNA immunoprecipitation (RIP)^38^. Magna RIP Kit (Millipore) was used to obtain cell lysates, and then immunoprecipitated with 50 μl of protein A/G magnetic beads and 5 μg of anti-FLAG antibody (Vigene Biosciences). A negative control was conducted by using normal rabbit IgG. After immobilizing with a magnet and washing with RIP Wash Buffer, the precipitated RNA was analyzed by qPCR. The final data were normalized to input to calculate the relative expression.

### Me-RIP-PCR

A previously described procedure was used for methylation RIP (Me-RIP)^38^. The Dynabeads mRNA Purification Kit (Invitrogen) was used to purify mRNA from the total RNA. Magna MeRIP^™^ m^6^A Kit (Millipore) was used to measure the change of m^6^A levels in mRNA according to the manufacturer’s protocol after RNA quality analyzing by NanoDrop 2000. After saving 0.5 μg of the mRNA as input, the remaining mRNA was used for m^6^A immunoprecipitation. After immunoprecipitation with Magna ChIP protein A/G Magnetic Beads and eluted twice with elution buffer, immunoprecipitated m^6^A RNAs were recovered by ethanol precipitation, and the RNA concentration was measured with NanoDrop 2000. Then immunoprecipitated m^6^A RNA was used as templates in RT-qPCR, as described above. RT-qPCR for FIP200 was conducted using the following primers: FIP200-1: forward 5′-AACTACTCTAACATTTGACACT-3′ and reverse 5′-TAAGGTCTGCCACAGTTT-3′, FIP200-2: forward 5′-ATCAGAATATGAAAGCCA-3′ and reverse 5′-CTAGCAATCATCTGGTCC-3′, and FIP200-3: forward 5′-TATGAAGGTAAACTTGAC-5′ and reverse 5′-TCTCCCTTCAATTTTTTA-3′. The housekeeping gene *HPRT1* was chosen as internal control since HPRT1 mRNA dose not have m^6^A peaks in m^6^A profiling data^22^.

### Flow cytometry

After the corresponding treatments, FITC-Annexin V Apoptosis Detection Kit (BD Pharmingen^TM^, 556547) was used according to the manufacturer’s instructions. Finally, samples stained with Annexin V and PI were quantitatively analyzed at 488 nm (emission wavelength) and 570 nm (excitation wavelength) by flow cytometry (BD FACSCalibur; BD Biosciences, San Jose, CA, USA).

### mRNA stability analysis

After treatment with 5 μg/ml actinomycin D (MedChem Express) to inhibit mRNA transcription, cells were collected at 0, 3, and 6 h to analyze mRNA levels and the rate of degradation. The total RNA was extracted and used for RT-qPCR. The degradation rate of RNA (k) was calculated using the equation: ${\mathrm{e}}^{ - kt} = N0/Nt$, *t* means the time after transcription inhibition and *k* represents the degradation rate, and *Nt* and *N*0 are the relative mRNA expression at time *t* and time 0. The RNA half-lifetime (*t*1/2) was calculated from the degradation rate as ${t}\frac{1}{2} = \frac{{ln2}}{k}$.

### Statistical analysis

Data are presented as the mean ± SD of at least three independent experiments. Statistical analyses were performed using GraphPad Prism 8 software (La Jolla, CA, USA). Differences between group means were evaluated with the Student *t* test or one-way ANOVA. *P* < 0.05 shows a statistical significance. *P < 0.05, **P < 0.01, and NS means no significance.

## Results

### Enhanced autophagy activity by BMSC coculture attenuates NPC apoptosis under compression

**Fig. 1:**
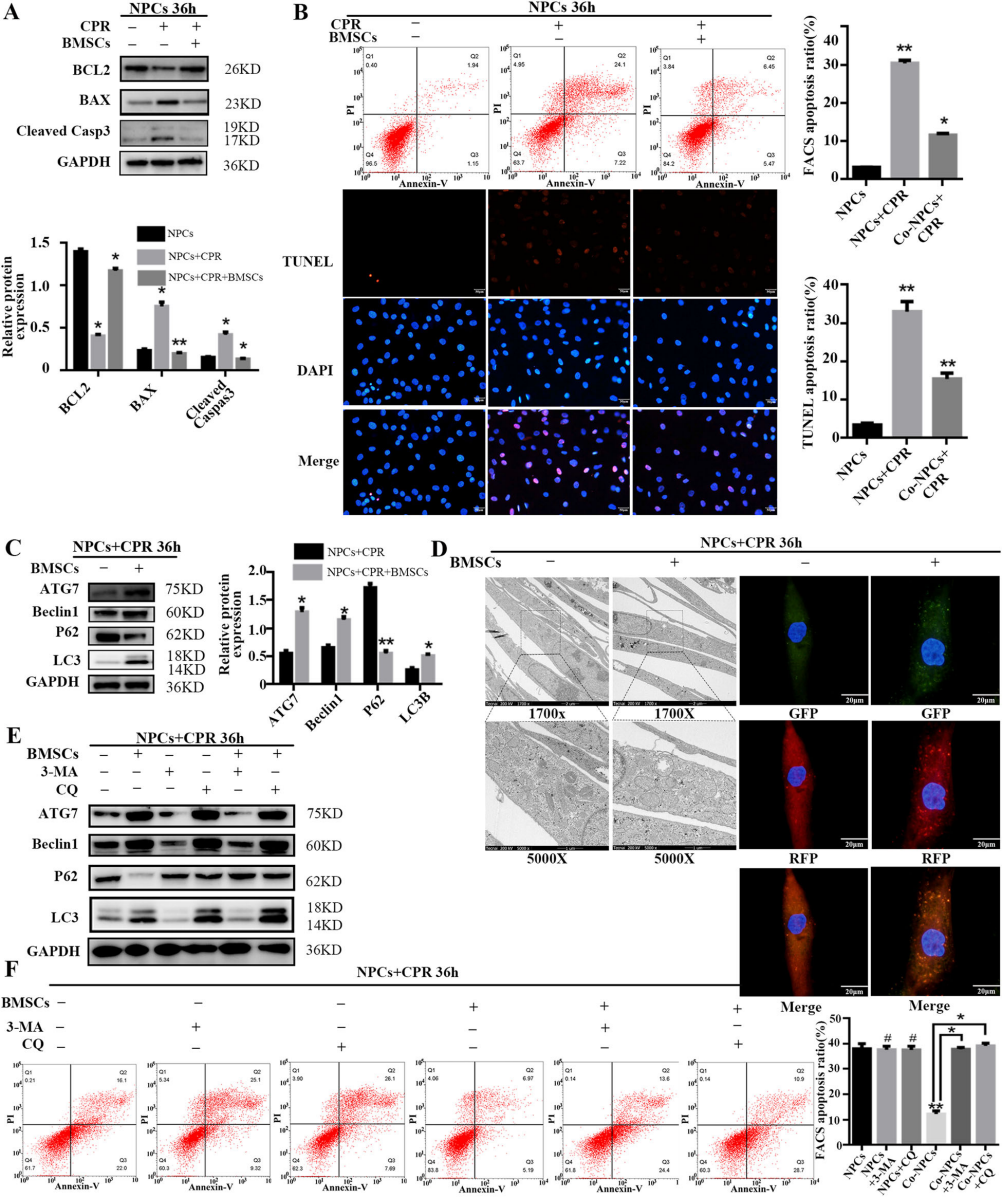
Enhanced autophagy activity by BMSC coculture attenuates NPC apoptosis under compression. **a** BCL2, BAX, and cleaved Caspase-3 protein levels in NPCs were determined by Western blot. GAPDH was used as a loading control. Expression of apoptosis-associated proteins was normalized to GAPDH; the results are presented as mean ± SD. Statistical analysis was conducted with the Student *t* test (*n* = 3, *P < 0.05, NPCs + CPR vs. NPCs; *P < 0.05, **P < 0.01, NPCs + CPR + BMSCs vs. NPCs + CPR). **b** FACS was performed to analyze the proportion of apoptotic NPCs in three groups. The sum of Annexin V^+^/PI^+^ cells (late apoptosis) and Annexin V^+^/PI^−^ (early apoptosis) was taken as the total apoptotic cells. TUNEL assay was performed to analyze the apoptosis of NPCs; TUNEL-positive cells (red) and DAPI-positive cells (blue) were merged (scale bar: 50 μm). The apoptosis ratio as detected by FACS analysis, and TUNEL staining was separately quantified as mean ± SD, as analyzed by unpaired Student *t* test (*n* = 3, **P < 0.01, NPCs + CPR vs. NPCs; *P < 0.05, **P < 0.01, NPCs + CPR + BMSCs vs. NPCs + CPR). **c** Atg7, Beclin1, P62, and LC3B protein levels in NPCs were determined by Western blot in NPCs under compression with or without BMSC coculture. GAPDH was used as a loading control. Protein expression levels were normalized to GAPDH; the results are presented as mean ± SD. Statistical analysis was conducted by unpaired Student *t* test (*n* = 3, *P < 0.05, **P < 0.01, NPCs + CPR + BMSCs vs. NPCs + CPR). **d** TEM was applied to observe autophagic vesicles in NPCs (scale bar: 2 μm [1700×]; scale bar: 1 μm [5000×]). Adenovirus harboring tandem fluorescent mRFP-GFP-LC3 was transfected into NPCs (Ad-LC3-NPCs) for 24 h, and cells were subjected to the corresponding treatments. Representative images of immunofluorescent NPCs expressing mRFP-GFP-LC3 are shown, and GFP (green), and mRFP dots (red) are merged (scale bar: 20 μm) (yellow dots in the merged image represent autophagosomes, and red-only dots in merged images represent autolysosomes). **e** Atg7, Beclin1, P62, and LC3B protein levels in NPCs were determined by Western blot in NPCs under compression in a coculture system, with the blockage of autophagy flux by 3-MA (10 μM) or chloroquine (CQ, 100 μM). GAPDH was used as a loading control. **f** FACS was performed to analyze the proportion of apoptotic NPCs in different groups. The sum of Annexin V^+^/PI^+^ cells (late apoptosis) and Annexin V^+^/PI^−^ (early apoptosis) cells was taken as the total apoptotic cells. The proportions of apoptotic cells as detected by FACS were compared by unpaired Student *t* test (*n* = 3, *P < 0.05, NPCs + BMSCs vs. NPCs; ^#^no significance, NPCs + 3-MA/CQ vs. NPCs; **P < 0.01, NPCs + BMSCs + 3-MA/CQ vs. NPCs + BMSCs).

NPCs under compression degenerate, and several studies have reported that injection of BMSCs can protect NPCs from apoptosis^1,2,39^. To observe the effect of compression on NPCs and the protective effect of BMSCs on NPCs when cocultured under 1.0-MPa compression for 36 h, Western blot, flow cytometry, and TUNEL assays were performed to examine the apoptotic rate of NPCs. The results show that the levels of cleaved Caspase-3 and the ratio of BAX/BCL2 were higher in the cocultured group than in the NPC control group (Fig. 1a). FACS and TUNEL analyses showed that the proportion of apoptotic cells was lower in the cocultured group (Fig. 1b). These data indicate that apoptosis of NPCs under compression could be reduced by BMSCs in a coculture system.

A previous study has shown that autophagy could reduce compression-induced apoptosis of human NPCs, so we wondered whether BMSCs could reduce apoptosis of NPCs under compression by stimulating autophagy. To investigate the role of autophagy in this process, we analyzed the protein levels of Atg7, Beclin1, P62, and LC3B by Western blot, and found that Atg7, Beclin1, and LC3B levels were increased in the coculture system, while P62 levels were decreased, indicating a significant increase in autophagic activity (Fig. 1c). The number of autophagy-associated vesicles increased, as observed by transmission electron microscopy (TEM), and yellow puncta accumulated after tandem fluorescent mRFP-GFP-LC3 transfection into NPCs (Fig. 1d), confirming that autophagic activity was increased. Two autophagy inhibitors, 3-methyladenine (3-MA) and chloroquine (CQ), were used to inhibit autophagy; as shown in Fig. 1e, autophagical activity was decreased, while the apoptotic rate was increased when an autophagy inhibitor was added to the medium, demonstrating that the effects of BMSCs on NPCs under compression were attenuated (Fig. 1e, f). These data suggest that the BMSCs reduce apoptosis of NPCs under compression by inducing autophagy.

### BMSC coculture enhances autophagy of NPCs under compression by regulating the ULK1 complex

**Fig. 2:**
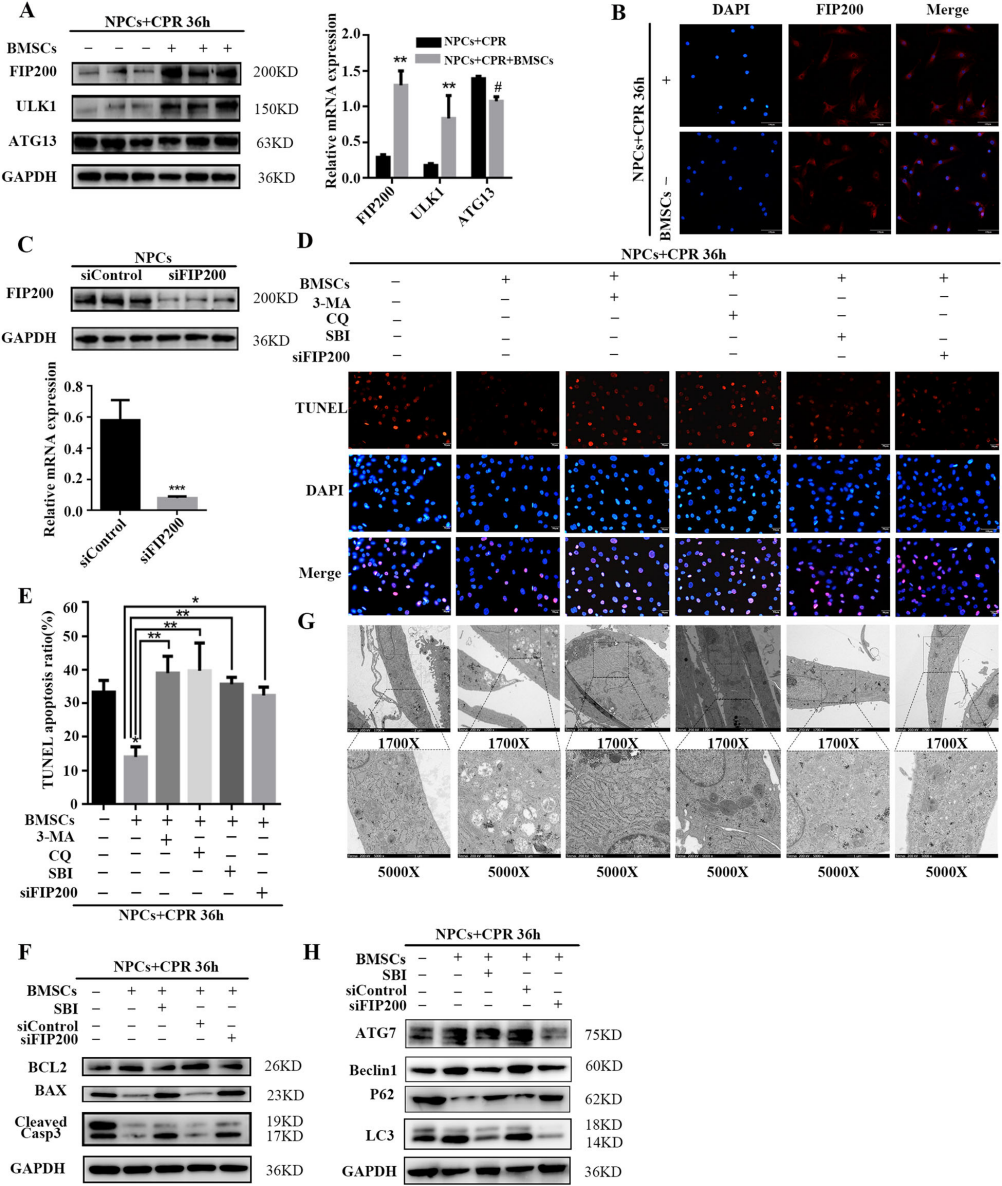
BMSC coculture enhances autophagy of NPCs under compression by regulating the ULK1 complex. **a** Three core components of ULK1 complex, that is, FIP200, ULK1, and Atg13 protein and transcripts levels in NPCs were determined by Western blot and RT-qPCR. GAPDH was used as a loading control. Expression levels were normalized to GAPDH; the results are presented as mean ± SD. Statistical analysis was conducted by unpaired Student’s *t* test (*n* = 3, *P < 0.05, **P < 0.01, ^#^no significance, NPCs + CPR + BMSCs vs. NPCs + CPR). **b** Immunofluorescence staining of FIP200 (red signal) and nucleus (blue signal) was analyzed by fluorescence microscopy (scale bar: 100 μm). The change in red fluorescence was significant. **c** *FIP200* knockdown efficiency was shown by Western blot and RT-qPCR. Statistical analysis was conducted by unpaired Student’s *t* test (*n* = 3, ***P < 0.001, siFIP200 vs. siControl). **d** Apoptosis of NPCs was analyzed by TUNEL assay after treatment with 3-MA, CQ, SBI-0206965 (10 μM), or siFIP200. TUNEL-positive cells (red) and DAPI-positive cells (blue) were merged (scale bar: 50 μm). **e** Percentage of apoptotic cells as detected by TUNEL staining. Statistical analysis was conducted by unpaired Student *t* test; the results are presented as mean ± SD (*n* = 3, *P < 0.05, BMSCs vs. blank; **P < 0.01, BMSCs + 3-MA/CQ/SBI vs. BMSCs; *P < 0.05, BMSCs + siFIP200 vs. BMSCs). **f** BCL2, BAX, and cleaved Caspase-3 protein levels in NPCs after treatment with SBI-0206965, siControl, or siFIP200 were determined by Western blot. GAPDH was used as a loading control. **g** Autophagic vesicles in NPCs were observed by TEM after treatment with 3-MA, CQ, SBI-0206965, or siFIP200 (scale bar: 2 μm [1700×]; scale bar: 1 μm [5000×]). **h** Levels of the autophagy-associated proteins Atg7, Beclin1, P62, and LC3B in NPCs under compression cocultured with BMSCs after treatment with SBI-0206965, siControl, or siFIP200, as determined by Western blot. GAPDH was used as a loading control.

The ULK1–FIP200–Atg13 complex can recruit other ATG proteins for autophagosome formation, regulate the dynamics of Atg9 vesicles, and activate the Vps34 PI(3)P kinase complex, which plays an essential role in the process of autophagy^40^. To elucidate the role of the ULK1 complex in this process, mRNA and protein levels of ULK1, FIP200, and Atg13 were measured by Western blot and RT-qPCR; in accordance with the higher autophagy activity, higher expression levels of ULK1 and FIP200 were observed in the coculture group, while Atg13 levels did not exhibit a significant difference (Fig. 2a). Immunofluorescence experiments also confirmed that the FIP200 expression levels were higher when NPCs were cocultured with BMSCs (Fig. 2b). A highly selective ULK1 kinase inhibitor, SBI-0206965, was used to inhibit ULK1 kinase activity^41^, and siRNA against FIP200 (siFIP200) was used to knock down FIP200 in NPCs to block the process of autophagy. First, knockdown efficiency was verified (Fig. 2c). Then apoptotic activity was measured by Western blot and Annexin-V/PI analysis, which showed that, compared with NPCs in the coculture system, SBI-0206965 and siFIP200 elevated the BAX:BCL2 ratio and cleaved Caspase-3 levels (Fig. 2d–f). Furthermore, the level of autophagic activity was evaluated; Western blot revealed that expression levels of Atg7, beclin1, and LC3B were decreased, and those of P62 were increased (Fig. 2h). TEM showed that inhibition of ULK1 and silencing of FIP200 decreased the number of autophagy-associated vesicles, indicating reduced autophagy activation (Fig. 2g). These results confirm that the process of autophagy was obstructed in the SBI-0206965 and siFIP200 groups, while apoptosis of cocultured NPCs was not reduced by BMSCs, highlighting the crucial role of the ULK1 complex in autophagy. The above results illustrate that BMSCs induce autophagy in NPCs under compression by regulating the ULK1–FIP200–Atg13 complex. Previous studies have reported that the protein levels of ULK1 were specifically upregulated by FTO-mediated m^6^A modification, thereby promoting the initiation of autophagy^35^. However, whether the epitranscriptomic modifications could regulate FIP200 levels and the process of autophagy remained unknown.

### ALKBH5 and the secondary m^6^A modification are involved in regulating NPC autophagy by BMSC coculture

**Fig. 3:**
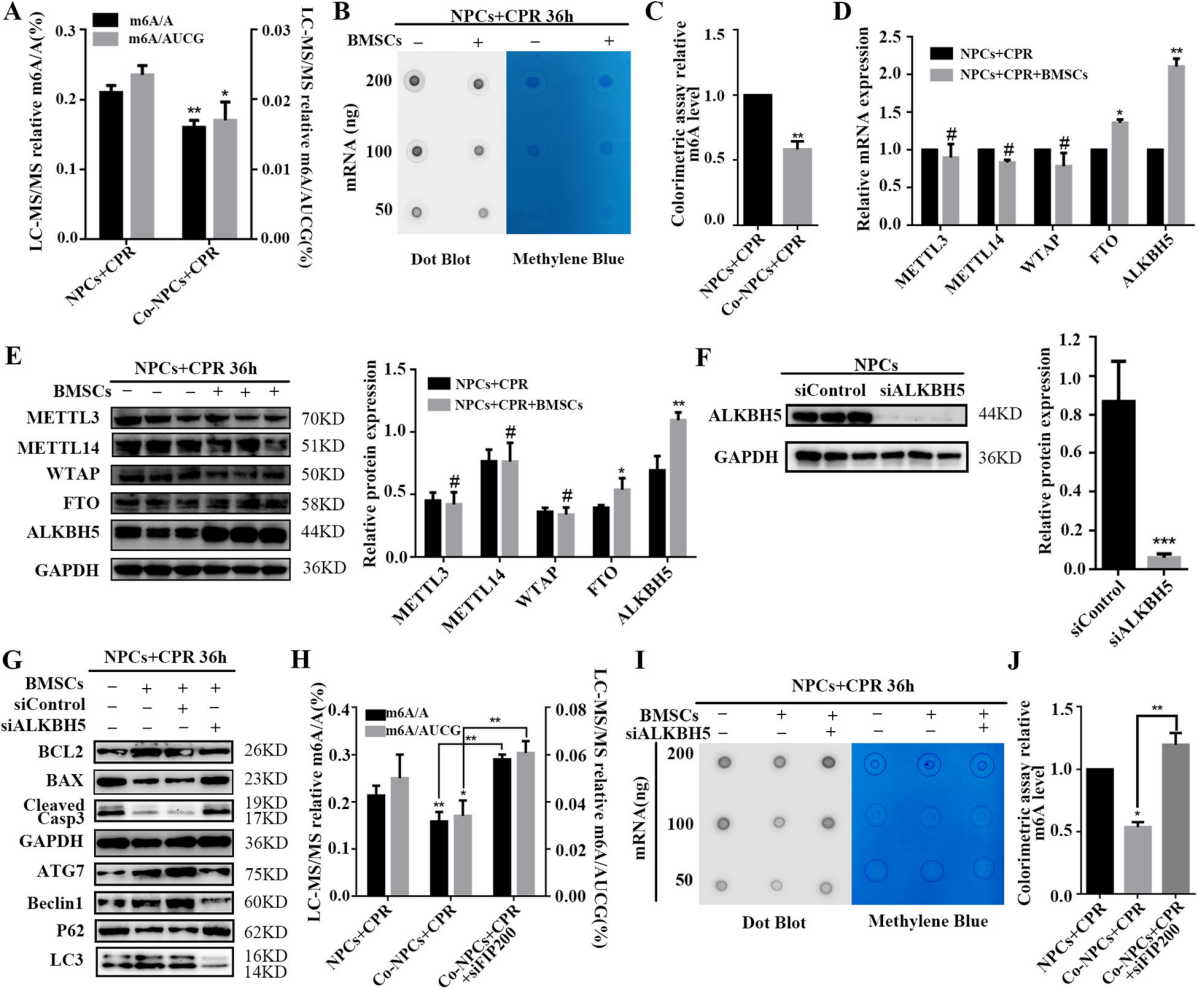
ALKBH5 and the secondary m^6^A modification are involved in regulating NPC autophagy by BMSC coculture. **a** The m^6^A/A ratio and m^6^A/AUCG ratio of total mRNA were determined by LC–MS/MS. Data are shown as mean ± SD. Statistical analysis was conducted by unpaired Student’s *t* test (*n* = 3, *P < 0.05, **P < 0.01; Co-NPCs + CPR vs. NPCs + CPR). **b** RNA dot blot was performed to analyze the m^6^A level of mRNA in NPCs with or without BMSC coculture. Methylene blue staining served as a loading control. **c** The relative m^6^A level in NPCs with or without BMSC coculture, as determined by colorimetric assay. Data are shown as mean ± SD. Statistical analysis was conducted by unpaired Student *t* test (*n* = 3, **P < 0.01, Co-NPCs + CPR vs. NPCs + CPR). **d** Relative mRNA levels of methyltransferases and demethylases were determined by RT-qPCR. The expression levels were normalized to the control group; the results are presented as mean ± SD. Statistical analysis was conducted by unpaired Student *t* test (*n* = 3, *P < 0.05, **P < 0.01, ^#^no significance, NPCs + BMSCs + CPR vs. NPCs + CPR). **e** Protein levels of the methyltransferases METTL3, METTL14, and WTAP, and the demethylases FTO and ALKBH5, were measured by Western blot. Protein levels were normalized to GAPDH; the results are presented as mean ± SD. Statistical analysis was conducted by unpaired Student’s *t* test (*n* = 3, *P < 0.05, **P < 0.01, NPCs + BMSCs + CPR vs. NPCs + CPR). **f** The *ALKBH5* knockdown efficiency by siALKBH5 was confirmed by Western blot. Expression levels were normalized to GAPDH; the results are presented as mean ± SD. Statistical analysis was conducted by unpaired Student *t* test (*n* = 3, **P < 0.01, siALKBH5 vs. siControl). **g** Protein levels of apoptosis- and autophagy-associated proteins after silencing of *ALKBH5* with or without BMSC coculture, as determined by Western blot. **h** m^6^A/A ratio and m^6^A/AUCG ratio of the total mRNA in three groups, as determined by LC–MS/MS. Data are shown as mean ± SD. Statistical analysis was conducted by unpaired Student’s *t* test (*n* = 3, *P < 0.05, **P < 0.01, Co-NPCs + CPR vs. NPCs + CPR; **P < 0.01, Co-NPCs + CPR + siFIP200 vs. Co-NPCs + CPR). **i** The m^6^A level of mRNA in NPCs with or without BMSC coculture upon *ALKBH5* knockdown was analyzed by RNA dot-blot analysis. Methylene blue staining served as a loading control. **j** Colorimetric assay to determine the relative m^6^A level in NPCs with or without BMSC coculture upon *ALKBH5* knockdown. Data are shown as mean ± SD. Statistical analysis was conducted by unpaired Student *t* test (*n* = 3, *P < 0.05, Co-NPCs + CPR vs. NPCs + CPR; **P < 0.01, Co-NPCs + CPR + siFIP200 vs. Co-NPCs + CPR).

In eukaryotic cells, m^6^A, one of the most prevalent epitranscriptomic modifications, has been widely investigated. The role of m^6^A modification in NPCs cocultured with BMSCs, was examined by LC–MS/MS. The results indicate that the m^6^A level of mRNAs isolated from cocultured NPCs was significantly lower than that in the corresponding control cells (Fig. 3a). Dot-blot and colorimetric assays were also performed, confirming these results, and highlighting that m^6^A may exert an essential function (Fig. 3b, c). Given that the methyltransferases METTL3, METTL14, and WTAP, and demethylases FTO and ALKBH5, are mainly responsible for the m^6^A levels, we measured the mRNA and protein expression levels of these components by RT-qPCR and Western blot. The results show that ALKBH5 is significantly upregulated when NPCs are cocultured with BMSCs, while the levels of other components did not exhibit large differences (Fig. 3d, e), suggesting that ALKBH5 mainly accounts for the lower level of m^6^A. Furthermore, to confirm the role of ALKBH5, knockdown experiments were carried out, and the efficiency of silencing was verified by Western blot (Fig. 3f). Next, apoptosis and autophagy in NPCs were assessed. Western blot results show that, upon ALKBH5 knockdown, the apoptosis-related protein cleaved Caspase-3 and the BAX:BCL2 ratio were upregulated, and autophagy-related proteins Atg7, Beclin1, and LC3 were downregulated while P62 was upregulated (Fig. 3g). As regards m^6^A modifications, LC–MS/MS analysis, dot blotting, and colorimetric assay results indicate a higher level of m^6^A modification in NPCs upon ALKBH5 knockdown (Fig. 3h–j). Our findings demonstrate that BMSC-induced autophagy in NPCs is dependent on ALKBH5-regulated m^6^A modification.

### BMSC coculture increases expression of FIP200 by m^6^A

**Fig. 4:**
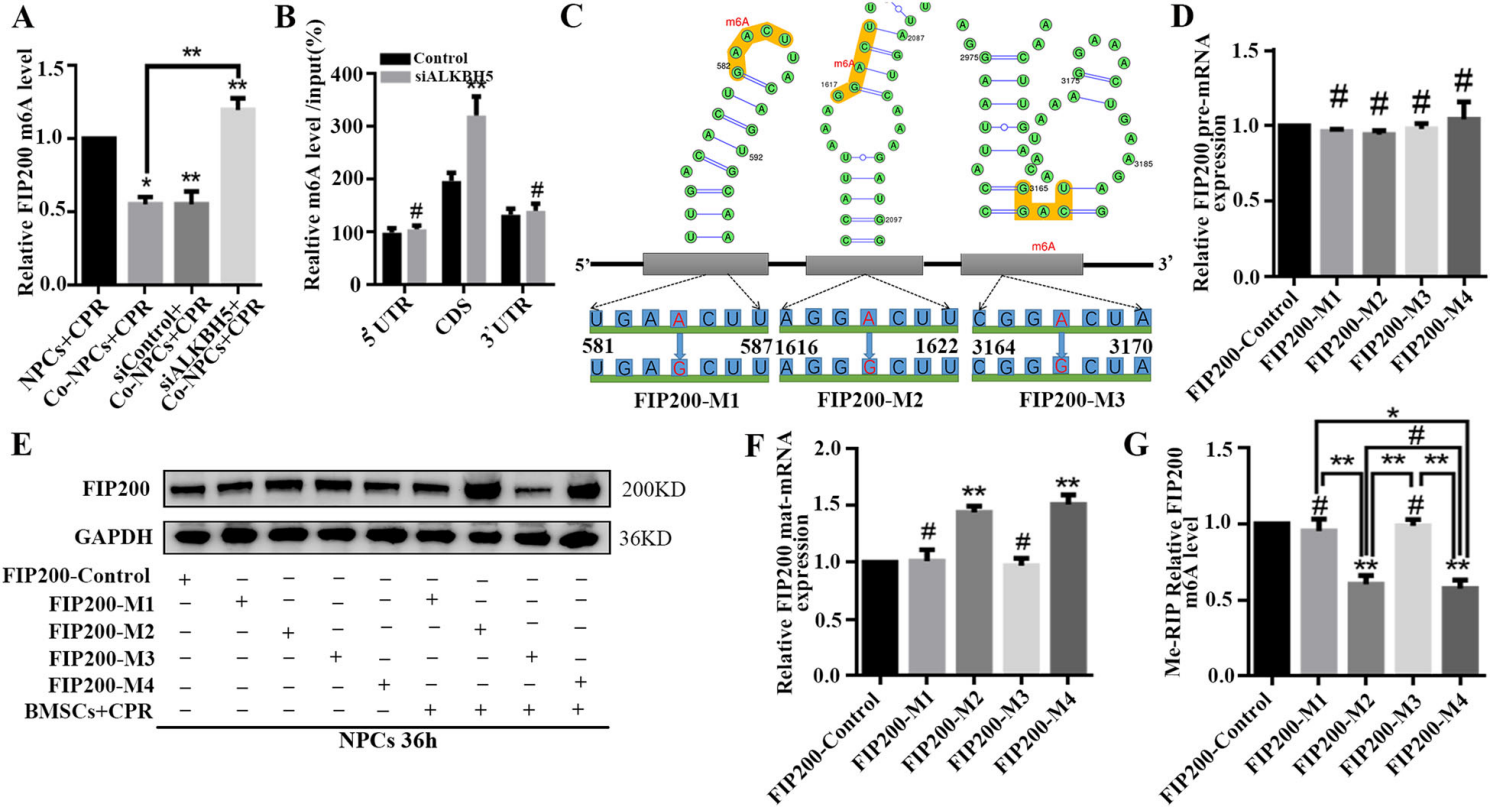
BMSC coculture increases expression of FIP200 by m^6^A. **a** The m^6^A level of *FIP200* transcripts was analyzed by Me-RIP-PCR. The m^6^A level was normalized to the input; the results are presented as mean ± SD. Statistical analysis was conducted by one-way ANOVA with Dunnet’s multiple comparison test (*n* = 3, *P < 0.05, **P < 0.01, Co-NPCs/siControl + Co-NPCs/siALKBH5 + Co-NPCs vs. NPCs; **P < 0.01, siALKBH5 + Co-NPCs vs. Co-NPCs). **b** Me-RIP-PCR analysis of 5′UTR, CDS, and 3′UTR fragments of *FIP200* mRNA in Control and siALKBH5 NPCs under compression cocultured with BMSCs; the results are presented as mean ± SD. Statistical analysis was conducted by unpaired Student *t* test (*n* = 3, **P < 0.01, ^#^no significance, siALKBH5 vs. siControl). **c** Predicted m^6^A sites and secondary structure in the CDS of *FIP200* mRNA. **d** Relative pre-mRNA levels of mutated *FIP200*, where three A residues were replaced with G. Data are shown as mean ± SD. Statistical analysis was conducted with one-way ANOVA with Dunnet’s multiple comparison test (*n* = 3, ^#^no significance, *FIP200-M1*/*M2*/*M3*/*M4* vs. *FIP200-Control*). **e** Protein expression levels of FIP200 and three FIP200 mutants where an A residue was replaced with G with or without BMSC coculture. **f** RT-qPCR was performed to analyze the mat-mRNA levels of control and its m^6^A site-mutated counterparts. Data are shown as mean ± SD. Statistical analysis was conducted with one-way ANOVA with Dunnet’s multiple comparison test (*n* = 3, **P < 0.01, ^#^no significance, *FIP200-M1*/*M2*/*M3*/*M4* vs. *FIP200-Control*). **g** Me-RIP-PCR analysis of m^6^A levels of mRNA of control FIP200 and its m^6^A site-mutated counterparts. Data are shown as mean ± SD. Statistical analysis was conducted with one-way ANOVA with Dunnet’s multiple comparison test (*n* = 3, **P < 0.01, ^#^no significance, *FIP200-M1*/*M2*/*M3*/*M4* vs. *FIP200-Control*; *n* = 3, **P < 0.01, *FIP200-M1*/*M3* vs. *FIP200-M2*; *P < 0.05, **P < 0.01, *FIP200-M1*/*M2* /*M4* vs. *FIP200-M4*).

Since ALKBH5-regulated m^6^A modification is required for the BMSC-induced increase in autophagy in NPCs, we hypothesized that ALKBH5 upregulates FIP200 in NPCs cocultured with BMSCs by m^6^A. Therefore, Me-RIP-PCR was performed to measure the m^6^A levels of FIP200 in NPCs with or without BMSC coculture. In the cocultured group, the methylation levels of FIP200 mRNA were decreased, which was reversed by ALKBH5 knockdown (Fig. 4a), indicating that ALKBH5-mediated demethylation of FIP200 is a critical step in the BMSC-induced activation of autophagy. To characterize the m^6^A modifications in FIP200 mRNA, an anti-m^6^A antibody was used to immunoprecipitate different fragments of mRNA isolated from NPCs. Me-RIP-PCR results show that the distribution of m^6^A in the coding region of FIP200 mRNA was higher than that in the 3′-UTR and 5′-UTR in control NPCs. In cocultured NPCs, the enrichment in m^6^A in the coding region of FIP200 was most significant in ALKBH5-silenced cells, suggesting that m^6^A methylation in the coding regions might be more significant than that in UTRs (Fig. 4b).

According to published studies, mRNA methylation reveals enrichment in coding regions, in 3′-UTRs, and near stop codons, which include an RRACU sequence motif^42^. To further investigate the precise sites of m^6^A modifications, the online tool SRAMP was used to predict the modification sites^43^. Multiple sites in the FIP200 coding region were strongly predicted to be modified, which is consistent with the reported studies using human HEK293T cells^21^ and HepG2 cells^20^. Next, the top three motifs in the coding region were chosen, and expression plasmids harboring single- or all three FIP200 point mutations in which the adenine residues embedded within the m^6^A motifs were replaced with guanine (*FIP200-M1*, *FIP200-M2, FIP200-M3*, and *FIP200-M4*; Fig. 4c), were constructed. Then the levels of precursor mRNA (pre-mRNA) and mature mRNA (mat-mRNA), and the levels of m^6^A modification in FIP200 of NPCs, were measured in the coculture system with compression. The results show that pre-mRNA levels were not significantly different between the three mutant forms and the control, while the mat-mRNA and protein expression levels of FIP200-M2 were significantly higher than those of M1 and M3 (Fig. 4d–f). Furthermore, Me-RIP-PCR analysis showed that FIP200-M2 mat-mRNA contained less m^6^A, indicating that the M2 mutation in FIP200 leads to lower methylation and higher mat-mRNA and protein levels, which are consistent with the results of the triple-mutated FIP200 (Fig. 4g). Therefore, we infer that 1619A is an essential adenosine group, as its methylation affects the degradation of FIP200 mRNA. These data confirm that lower methylation of FIP200 mRNA is correlated with higher levels of FIP200, promoting autophagy.

### m^6^A regulates mRNA stability of FIP200

**Fig. 5:**
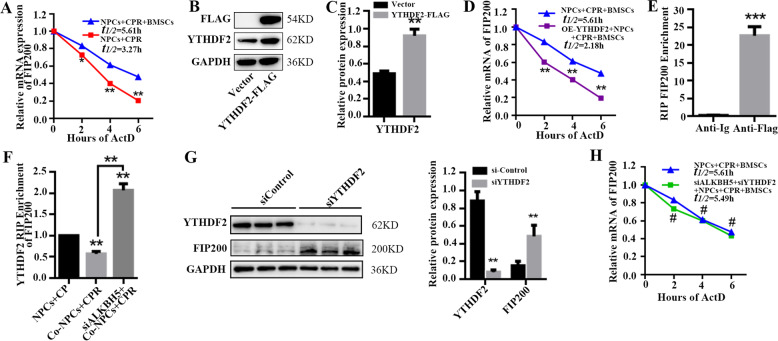
m^6^A regulates mRNA stability of FIP200. **a** The mRNA levels of FIP200 after treatment with actinomycin D were measured by RT-qPCR to analyze the stability of FIP200 in NPCs cocultured with BMSCs. mRNA half-life (*t*_1/2_) was calculated, and statistical analysis was conducted with the Student *t* test (*n* = 3, ***P < 0.001, NPCs + CPR + BMSCs vs. NPCs + CPR). **b** FLAG and YTHDF2 levels in cells transfected with control and YTHDF2-FLAG plasmid were analyzed by Western blot. GAPDH was used as loading control. **c** YTHDF2 protein expression levels were normalized to GAPDH; the results are presented as mean ± SD. Statistical analysis was conducted by unpaired Student *t* test (*n* = 3, **P < 0.01, YTHDF2-FLAG vs. Vector). **d** The stability of *FIP200* in YTHDF2-overexpressing NPCs cocultured with BMSCs was analyzed by RT-qPCR. mRNA half-life (*t*_1/2_) was calculated, and data are shown as mean ± SD. Statistical analysis was conducted with the Student *t* test (*n* = 3, ***P < 0.001, OE-YTHDF2 + NPCs + CPR + BMSCs vs. NPCs + CPR + BMSCs). **e** The interaction of FIP200-FLAG in NPCs transfected with control or YTHDF2-FLAG was analyzed by RIP-qPCR. FIP200-FLAG enrichment was normalized to input; the results are presented as mean ± SD. Statistical analysis was conducted by unpaired Student *t* test (*n* = 3, ***P < 0.001, Anti-Flag vs. Anti-IgG). **f** The interaction of FIP200 with YTHDF2 in control and *ALKBH5* knockdown NPCs with or without BMSCs cocultured was analyzed by RNA IP. Data are shown as mean ± SD. Statistical analysis was conducted with one-way ANOVA with Dunnet’s multiple comparison test (*n* = 3, **P < 0.01, Co-NPCs/siALKBH5 + Co-NPCs vs. NPCs; **P < 0.01, siALKBH5 + Co-NPCs vs. Co-NPCs). **g** YTHDF2 and FIP200 protein levels in control, and *YTHDF2*-knockdown NPCs after BMSC coculture under compression for 36 h, as measured by Western blot. Protein expression levels were normalized to GAPDH; the results are presented as mean ± SD. Statistical analysis was conducted by unpaired Student *t* test (*n* = 3, **P < 0.01, siYTHDF2 vs. siControl). **h** *FIP200* mRNA levels after treatment with actinomycin D were analyzed by RT-qPCR to examine the stability of *FIP200* mRNA in *ALKBH5* and *YTHDF2*-knockdown NPCs cocultured with BMSCs. Data are shown as mean ± SD. mRNA half-life (*t*_1/2_) was calculated, and statistical analysis was conducted with the Student *t* test (*n* = 3, ^#^no significance, siALKBH5 + siYTHDF2 + NPCs vs. NPCs).

YTHDF2, the first modification-specific RNA-binding protein or “reader” identified, can increase the turnover of m^6^A-modified mRNA by promoting colocalization with degradation factors^20^. Furthermore, in HeLa cells, functional clustering analysis of YTHDF2 targets was conducted, and the top functions identified were the molecular function “Gene Expression and RNA Transcription” and the cellular function “Cell Death and Survival”^22^. Due to the negative correlation between m^6^A levels and protein abundance of FIP200, we hypothesized that FIP200 levels are regulated by m^6^A through YTHDF2-mediated mRNA degradation. To further confirm that the stability of FIP200 mRNA is regulated by YTHDF2, FIP200 mRNA levels were measured, and the stability of FIP200 mat-mRNA in NPCs was analyzed after using the transcription inhibitor actinomycin D. The results show that mRNA levels of FIP200 in NPCs cocultured with BMSCs were higher than in those without coculture after actinomycin D was added for 2, 4, and 6 h, indicating that FIP200 transcripts with fewer m^6^A modifications in NPCs in the coculture system are more stable (Fig. 5a). We then generated a FLAG-tagged YTHDF2 plasmid (YTHDF2-FLAG) to investigate whether FIP200 is one of the target genes of YTHDF2. First, we confirmed that YTHDF2-FLAG was significantly expressed by Western blot (Fig. 5b, c). FIP200 mRNA levels in YTHDF2-overexpressing NPCs cocultured with BMSCs were decreased after treatment with actinomycin D, demonstrating that overexpression of YTHDF2 in NPCs could abolish the stabilizing effect of BMSCs (Fig. 5d). We showed that FIP200 interacts with YTHDF2-FLAG by RIP-qPCR using an anti-FLAG antibody (Fig. 5e). Moreover, the number of FIP200 transcripts high in m^6^A, which can be recognized by YTHDF2, in NPCs was lower in the coculture than in the control group, but was significantly increased upon transfection with siRNA against ALKBH5 (siALKBH5) (Fig. 5f). Consistently, knockdown of the prodegradation “reader” YTHDF2 with siRNA against YTHDF2 (siYTHDF2) could increase the FIP200 protein levels (Fig. 5g). Moreover, loss of ALKBH5 could downregulate the stability of FIP200 transcripts, as verified by the lower FIP200 mRNA levels upon actinomycin D treatment (Fig. 5h), confirming that YTHDF2 affects mRNA stability in a m^6^A-dependent manner. These results prove that the expression of ALKBH5 reduces m^6^A modification of FIP200 mRNA, leading to a decrease in YTHDF2-mediated mRNA degradation and an increase in FIP200 levels.

### Silencing of YTHDF2 partially restores FIP200 levels and rescues autophagy in ALKBH5-depleted NPCs cocultured with BMSCs

**Fig. 6:**
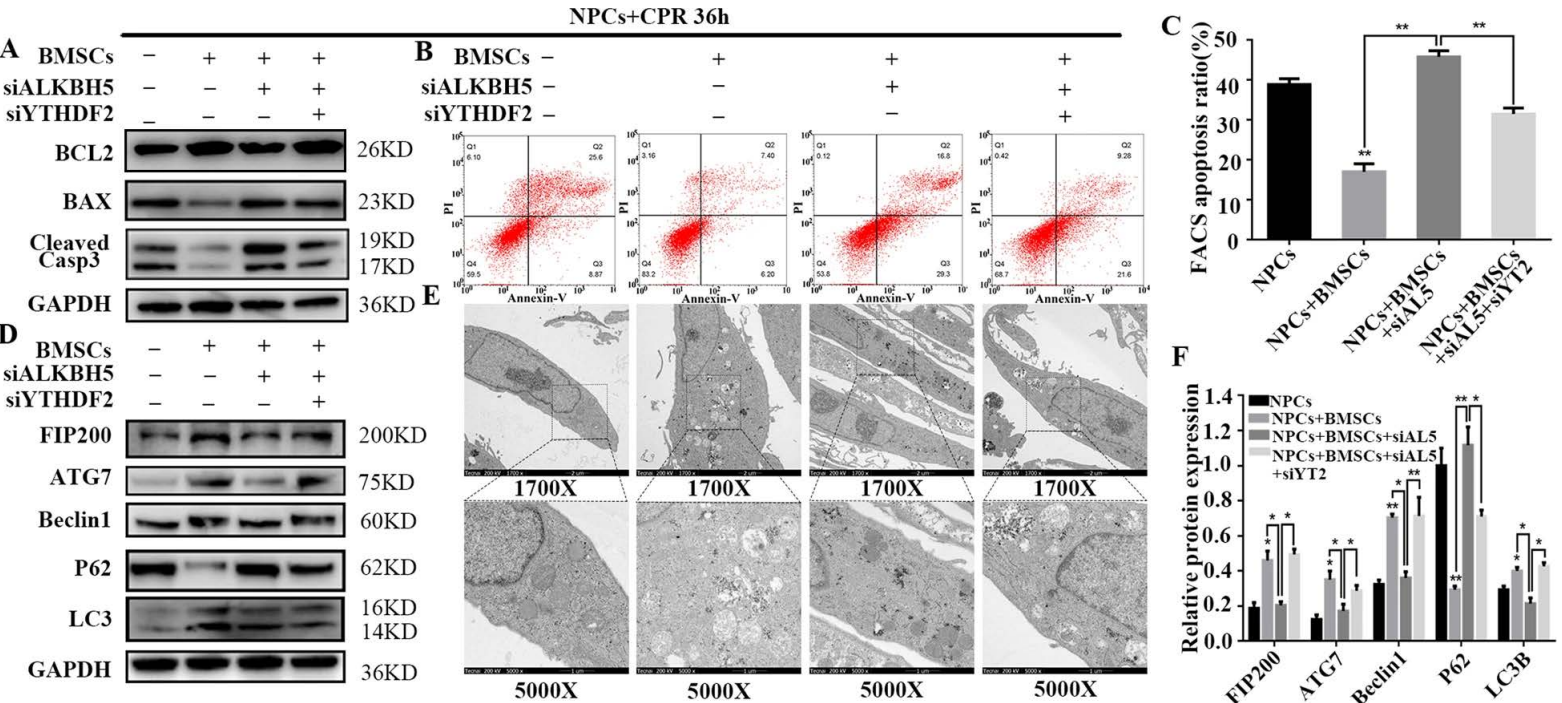
Silencing of YTHDF2 partially restores FIP200 levels and rescues autophagy in ALKBH5-depleted NPCs cocultured with BMSCs. **a** BCL2, BAX, and cleaved Caspase-3 protein levels in *ALKBH5* or *YTHDF2*-knockdown NPCs under compression with or without BMSC coculture were determined by Western blot. GAPDH was used as a loading control. **b**, **c** The percentage of apoptotic ALKBH5 or YTHDF2-knockdown NPCs with or without BMSC coculture, as determined by FACS analysis. The sum of Annexin V^+^/PI^+^ cells (late apoptosis) and Annexin V^+^/PI^–^ (early apoptosis) cells was taken as the total apoptotic cells. The percentage of apoptotic cells as detected by FACS is presented as mean ± SD. Statistical analysis was conducted by one-way ANOVA with Dunnet’s multiple comparison test (*n* = 3, **P < 0.01, NPCs + BMSCs vs. NPCs; *P < 0.05, **P < 0.01, BMSCs/siALKBH5 + siYTHDF2 + BMSCs vs. siALKBH5 + BMSCs). **d** Expression levels of the autophagy-associated proteins FIP200, Atg7, Beclin1, P62, and LC3 in *ALKBH5* or *YTHDF2*-knockdown NPCs under compression with or without BMSC coculture were determined by Western blot. GAPDH was used as a loading control. **e** TEM was applied to observe autophagic vesicles in *ALKBH5* or *YTHDF2*-knockdown NPCs with or without coculture (scale bar: 2 μm [1700×]; scale bar: 1 μm [5000×]). **f** Expression levels of autophagy-associated proteins were normalized to GAPDH; the results are presented as mean ± SD. Statistical analysis was conducted with one-way ANOVA with Dunnet’s multiple comparison test (*n* = 3, *P < 0.05, **P < 0.01, NPCs + BMSCs vs. NPCs; *P < 0.05, **P < 0.01, NPCs + BMSCs/siALKBH5 + siYTHDF2 + NPCs + BMSCs vs. siALKBH5 + NPCs + BMSCs).

To confirm the role of YTHDF2-mediated degradation of m^6^A-modified FIP200 in the interplay between autophagy and apoptosis, YTHDF2 was knocked down in ALKBH5-silenced NPCs, which were then cocultured with BMSCs and exposed to compression. The percentage of apoptotic cells was analyzed and compared with that in the ALKBH5-silenced group. The protein levels of cleaved Caspase-3 and the ratio of BAX:BCL2 were decreased, and flow cytometry indicated a lower percentage of apoptotic NPCs when both YTHDF2 and ALKBH5 were silenced (Fig. 6a–c). The results suggest that apoptosis of ALKBH5-depleted NPCs could be partially rescued by YTHDF2 silencing upon coculture with BMSCs under compression. Moreover, we found that the expression of FIP200 and other autophagy components was reversed upon knockdown of YTHDF2, and TEM revealed a higher number of autophagy-associated vesicles (Fig. 6d–f), evidencing the recovery of FIP200 expression and autophagic activity. Collectively, these results show that knockdown of YTHDF2 partially restores FIP200 expression and rescues autophagy in ALKBH5-depleted NPCs cocultured with BMSCs, thus attenuating apoptosis of NPCs.

## Discussion

**Fig. 7:**
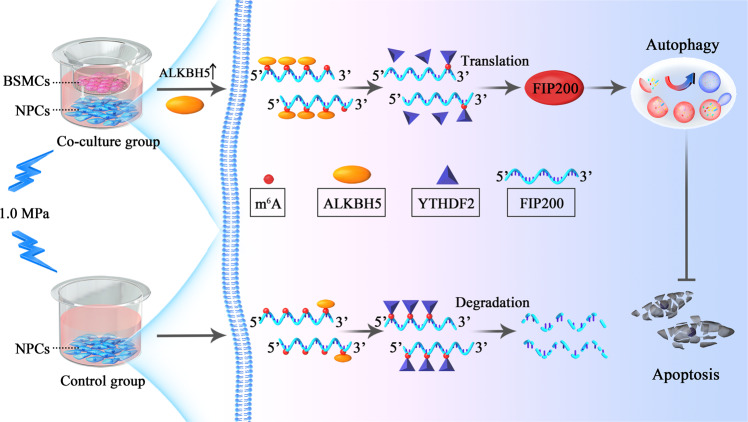
Schematic overview of the role of BMSCs in alleviating apoptosis of NPCs by m^6^A modification of mRNAs that play a role in autophagy. BMSCs increase ALKBH5 expression in NPCs when cocultured with BMSCs, thus decreasing m^6^A modification of *FIP200* mRNA. A lower level of methylation of *FIP200* transcripts results in less YTHDF2-mediated *FIP200* mRNA degradation, increasing *FIP200* levels, enhancing autophagy, and reducing compression-induced apoptosis in NPCs.

Several studies have suggested that m^6^A modification of mRNA regulates autophagy and cell fate by various mechanisms^35,44–46^. In this study, we identified a crucial role of m^6^A in the regulation of FIP200 levels, and thereby autophagy and apoptosis, in NPCs under compression, are cocultured with BMSCs. Briefly, when NPCs under compression increase significantly when they are cocultured with BMSCs, autophagic activity increases significantly. Upon treatment with an autophagy inhibitor (3-MA or CQ), the effects of BMSCs on NPCs were abolished. ULK1 and FIP200, two key components of the ULK1 complex, were upregulated in NPCs when cocultured with BMSCs; upon treatment with a specific inhibitor of ULK1 kinase (SBI-0206965) or siFIP200, the effects of BMSCs on autophagic activity were inhibited. A previous study has reported that m^6^A can upregulate ULK1 protein levels and control autophagy. To investigate whether the variation in FIP200 levels during autophagy could be regulated by m^6^A modification^35^, Me-RIP was performed; the results indicate that the level of m^6^A modification was lower in the coculture system, implying that m^6^A modification participates in the regulation of autophagy in NPCs. Of the main methyltransferases and demethylases, ALKBH5, exhibited the largest difference between control NPCs and the coculture group, and gain- and loss-of-function studies revealed that ALKBH5 could regulate FIP200 levels via demethylating one adenosine group in the coding region of the mRNA, providing protection against YTHDF2-mediated degradation. Deletion of ALKBH5 could attenuate the effect of BMSCs on NPCs under compression, which could be reversed by knockdown of YTHDF2, further indicating that BMSC-mediated upregulation of ALKBH5 leads to demethylation of FIP200 mRNA, resulting in less YTHDF2-mediated degradation, thus promoting autophagic flux and protecting NPCs from apoptosis (Fig. 7).

Several studies have pointed out the regulatory role of m^6^A in mRNA degradation^22,47,48^. In cooperation with “readers,” the dynamic m^6^A modification is recognized by binding proteins, which affect the stability and lifetime of mRNAs. A recent investigation conducted in human fibroblasts indicated that genes stimulated by interferon after virus infection could be induced once METTL3 and YTHDF2 were repressed, which showed that the m^6^A modification of murine IFNβ mRNA could accelerate its degradation^49^. During the process from naive pluripotency toward differentiation, and during pluripotency maintenance of hPSCs, the absence of m^6^A leads to a direct increase in stability of mRNAs, including transcripts encoding developmental regulators, and those of prominent naive pluripotency regulators, impair embryonic stem cell differentiation, and stabilize the pluripotent state, demonstrating that m^6^A modification could fine-tune the state of stem cells^38,47,50,51^. Besides, ALKBH5-mediated demethylation of NANOG transcripts mediates breast cancer stem cell enrichment in the hypoxic tumor microenvironment, and higher stability of TFEB mRNA in cardiomyocytes with fewer m^6^A modifications due to ALKBH5 reverses H/R-mediated TFEB mRNA degradation, and protects cardiomyocytes from apoptosis^34,44^. Several studies have indicated that transcripts with more m^6^A modifications tend to be less stable, mainly due to the relocation of such mRNAs by YTHDF2 to RNA degradation complexes, and functional clustering analysis of YTHDF2 targets in HeLa cells was conducted to identify the top functions, which are the molecular function “Gene Expression and RNA Transcription” and the cellular function “Cell Death and Survival”^22^. In this study, we discovered that NPCs under compression could be rescued from apoptosis by BMSCs through ALKBH5-mediated protection from degradation of FIP200. However, we did not exclude potential functions or binding activities of other “readers,” like YTHDF1, YTHDF3, and YTHDC2, to other m^6^A-modified transcripts and their effects on autophagy, which will require further investigations.

NPC apoptosis induced by mechanical stimuli mainly accounts for IVD degeneration. The use of BMSCs to reverse the process of degeneration and regenerate more NPCs is a promising strategy that has been validated in mouse and goat models^52,53^. Moreover, clinical trials using BMSCs for the treatment of chronic low back pain caused by IVD degeneration have been conducted, and long-term results indicate that injection of BMSCs into IVDs is well tolerated and provides substantial improvement in pain and function^2^. Furthermore, recent studies confirmed that NPCs can be protected from apoptosis through induction of autophagy during the process of IVD degeneration^11,54,55^. In the compression-induced model, BMSCs could inhibit the mitochondrial pathway to protect NPCs from apoptosis^39^. In this study, we used compression of 1.0 MPa for 36 h, which has been confirmed to be consistent with the pressure in intervertebral discs in a standing position^56^, to investigate the protective effect of BMSCs on compression-induced NPC apoptosis.

FIP200 is an essential subunit of the ULK1 complex, which acts at the early stages of autophagosome formation. Loss of FIP200-induced autophagy downregulation was able to decrease bam mutant stem cell niche occupancy, indicating that specifically activating autophagy through FIP200 could provide a new therapeutic strategy^57^. Furthermore, the tumor suppressor protein p53 can inhibit autophagy by directly interacting with FIP200, suppressing tumor progression^58^. Besides, a recent study proved that a claw-shaped domain of FIP200 directly interacts with a region of SQSTM1/P62 containing its LIR motif to induce autophagosome formation^59^. FIP200 levels were decreased in NPCs under compression, and BMSCs could recover FIP200 levels and autophagy, thus reducing the apoptosis ratio. Recent reports have shown that in different cells, the expression of several core components of autophagy flux, that is, ULK1, Atg5, and Atg7 could be regulated in a m^6^A-dependent manner, thus affecting the process of autophagy^35,46^. Me-RIP revealed that, in the coculture system, FIP200 transcripts were modified with less m^6^A, due to the upregulation of ALKBH5, resulting in less YTHDF2-mediated FIP200 mRNA degradation. However, it should be noted that the question whether other methyltransferases and demethylases could modify the methylation of other transcripts and regulate autophagy still requires more study, and the molecular mechanisms by which BMSC coculture regulates ALKBH5 in NPCs are worthy of further study.

In summary, our research revealed that apoptosis of NPCs under compression could be inhibited by coculturing with BMSCs, which regulates the process of autophagy in NPCs by upregulating ALKBH5, which protects FIP200 mRNA from YTHDF2-mediated degradation. Our data clarify the mechanism of interaction of NPCs and BMSCs, providing a new theoretical basis for the application of BMSCs to reverse IVD degeneration.
