# Correction: Bone-derived mesenchymal stem cells alleviate compression-induced apoptosis of nucleus pulposus cells by N6 methyladenosine of autophagy

**DOI:** 10.1038/s41419-025-08383-5

**Published:** 2026-03-24

**Authors:** Gaocai Li, Yu Song, Zhiwei Liao, Kun Wang, Rongjin Luo, Saideng Lu, Kangcheng Zhao, Xiaobo Feng, Hang Liang, Liang Ma, Bingjin Wang, Wencan Ke, Huipeng Yin, Shengfeng Zhan, Shuai Li, Xinghuo Wu, Yukun Zhang, Cao Yang

**Affiliations:** Department of Orthopaedics, Union Hospital, Tongji Medical College, Huazhong University of Science and Technology, Wuhan, 430022 China

Correction to: *Cell Death & Disease* 10.1038/s41419-020-2284-8, published online 06 February 2020

In this article, the image in upper panel of BCL2 in Figure 6A was misused.

Incorrect Fig. 6
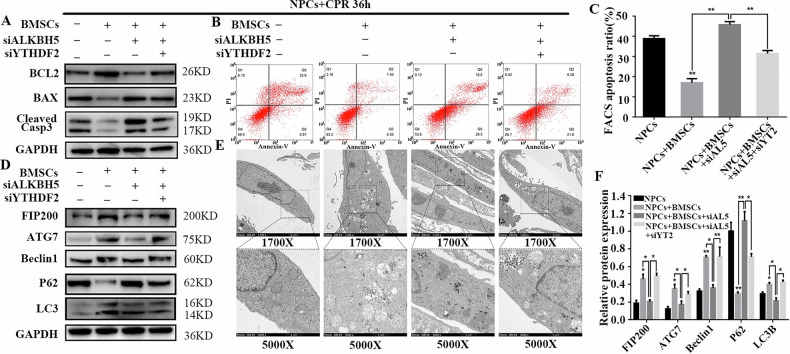


Correct Fig. 6
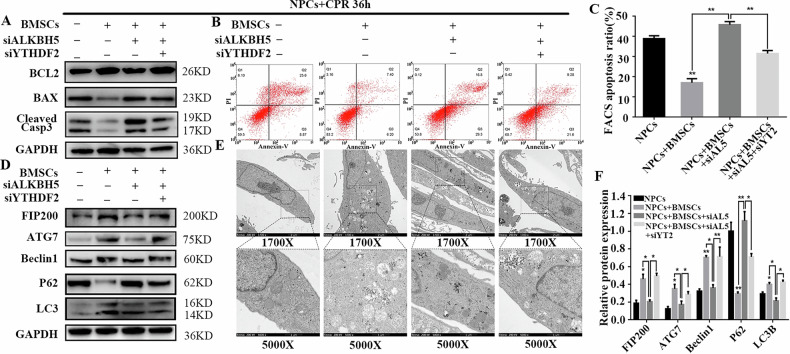


## Supplementary information

Original data

