# Supplementary figures and images for: Correction: Bone-derived mesenchymal stem cells alleviate compression-induced apoptosis of nucleus pulposus cells by N6 methyladenosine of autophagy
Source: Cell Death Dis. 2026 Mar 24;17(1):279. doi: 10.1038/s41419-025-08383-5 (PMC13013695; doi:10.1038/s41419-025-08383-5)

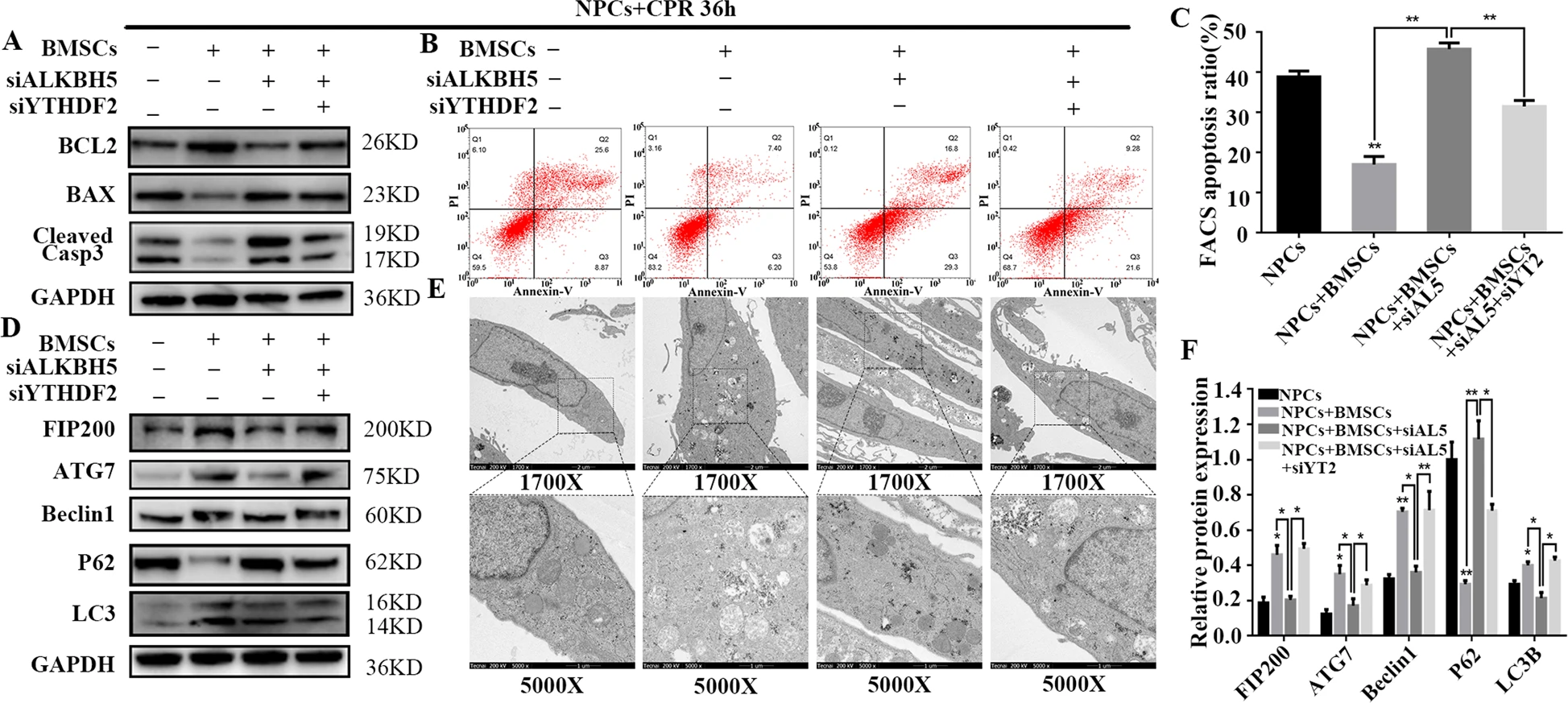

Supplement: Supplementary file 1 — Original data [file 41419_2025_8383_MOESM1_ESM.tif]
